# Hemoglobin Vesicles prolong the time to circulatory collapse in rats during apnea

**DOI:** 10.1186/s12871-017-0338-y

**Published:** 2017-03-14

**Authors:** Yusuke Naito, Hiromi Sakai, Satoki Inoue, Masahiko Kawaguchi

**Affiliations:** 1 Department of Anesthesiology, Nara Medical University, 840 Shijo-cho, Kashihara, Nara 634-8522 Japan; 2 Department of Chemistry, Nara Medical University, 840 Shijo-cho, Kashihara, Nara Japan

**Keywords:** Hemoglobin Vesicles, Artificial red blood cells, Apnea, Hypoxia

## Abstract

**Background:**

Hemoglobin vesicles (HbV) are hemoglobin-based oxygen carriers manufactured by liposome encapsulation of hemoglobin molecules. We hypothesised that the infusion of oxygenated HbV could prolong the time to circulatory collapse during apnea in rats.

**Methods:**

Twenty-four Sprague-Dawley rats were randomly divided into four groups (Air, Oxy, NS and HbV). The rats were anaesthetized with isoflurane and the trachea was intubated using 14-gauge intravenous catheters. Rats in the Air group were mechanically ventilated with 1.5% isoflurane in room air, and those in other groups received 1.5% isoflurane in 100% oxygen. Mechanical ventilation was withdrawn 1 min after the administration of rocuronium bromide to induce apnea. After 30 s, 6 mL saline and HbV boluses were infused at a rate of 0.1 mL/s in the NS and HbV groups, respectively. Circulatory collapse was defined as a pulse pressure < 20 mmHg and the time to reach this point (PP_20_) was compared between the groups. The results were analysed via a one-way analysis of variance and post-hoc Holm–Sidak test.

**Results:**

PP_20_ times were 30.4 ± 4.2 s, 67.5 ± 9.7 s, 95 ± 17.3 s and 135 ± 38.2 s for the Air (ventilated in room air with no fluid bolus), Oxy (ventilated with 100% oxygen with no fluid bolus), NS (ventilated with 100% oxygen with a normal saline bolus), and HbV (ventilated in 100% oxygen with an HbV bolus) groups, respectively, and differed significantly between the four groups (*P* = 0.0001). The PP_20_ times in the HbV group were significantly greater than in the Air (*P* = 0.0001), Oxy (*P* = 0.007) and NS (*P* = 0.04) groups.

**Conclusion:**

Infusion of oxygenated HbV prolongs the time to circulatory collapse during apnea in rats.

**Electronic supplementary material:**

The online version of this article (doi:10.1186/s12871-017-0338-y) contains supplementary material, which is available to authorized users.

## Background

The situation ‘cannot ventilate, cannot intubate (CVCI)’ during the induction of anaesthesia is one of the most serious complications anaesthesiologists can encounter. In some cases, supraglottic airway devices may help to maintain oxygenation, but otherwise, the reinitiation of spontaneous ventilation by waking the patient, or the initiation of emergency intubation pathways, should be considered [1]. Even after the introduction of fast-acting drugs and Sugammadex, the time required for the reestablishment of spontaneous breathing is often longer than expected [2]. Emergency intubation pathways commonly involve invasive airway access that require at least a few minutes of preparation. These techniques are also highly invasive, and each carries a substantial risk of technical complications [3].

Hemoglobin vesicles (HbV) are a type of cellular Hb-based oxygen carrier (HBOC) manufactured by the encapsulation of a purified, concentrated Hb solution using liposomes [4]. Moreover, HbV was developed as an alternative to red blood cell (RBC) transfusion and has exhibited efficacy as components of the resuscitative fluid used to treat haemorrhagic shock in emergency situations using animal models [5, 6]. The absence of a blood type antigen and infectious viruses, the small particle size required for penetration via constricted vessels through which red blood cells cannot penetrate, and the stability for long-term storage at room temperature are also important advantages of HbV [7]. In practice, HbV is stored in a deoxygenated state, but is rapidly oxygenated and administered within extremely short time periods. Therefore, we hypothesised that the infusion of oxygenated HbV could maintain oxygenation in critical situations (e.g. CVCI) after the induction of anaesthesia. Accordingly, we tested whether the administration of HbV could prolong the time to circulatory collapse during apnea in a rat model.

## Methods

All experiments were conducted using 24 male Sprague-Dawley rats (300–330 g, 10 weeks old). All animals were purchased from SLC Inc., Shizuoka, Japan. The animals were housed on a bed of cellulose paper in a ventilated, temperature controlled, specific-pathogen-free environment with a 12-h light-dark cycle. The animals were provided with access to food and water *ad libitum*. All experimental protocols were reviewed by the Committee on the Ethics of Animal Experiments at our University and were conducted in accordance with the Guidelines for Animal Experiments issued by the Nara Medical University and with law no. 105 (Act on Welfare and Management of Animals) issued by the Japanese government. The ethical guidelines conformed to the guiding principles issued by the National Academy of Science.

### Pilot study to define circulatory collapse

To perform a thorough investigation, we first sought to define the criteria for circulatory collapse. There are previous reports of apnea in rat models; however, there is no consensus on the definition of circulatory collapse in these reports [8, 9]. Moreover, a number of previous studies have used the mean arterial pressure or systolic blood pressure to define circulatory collapse [10, 11]; however, these criteria were not used in the present study as volume infusions lead to a 30% increase in the total blood volume of the rats, likely resulting in an increased mean arterial and systolic blood pressure. Instead, we used the pulse pressure to define circulatory collapse in our study. We assessed a range of pulse pressures (5, 10, 15, 20 and 25 mmHg) as surrogates of circulatory collapse. The same equipment and drugs described in the experimental methodology were used in this pilot study. In brief, five rats were orally intubated after the induction of anaesthesia via the inhalation of 5% isoflurane in 100% oxygen. The trachea of the rats was then intubated using 14-gauge intravenous catheters. Mechanical ventilation was initiated with 1.5% isoflurane in room air at 1 L/min and oxygen at 1 L/min to maintain an FIO_2_ of 0.6. Adequate anaesthesia was confirmed by the absence of the pedal withdrawal reflex in response to tail pinching. Polyethylene catheters were introduced into the tail arteries to monitor the arterial blood pressure. Polyethylene catheters were also introduced into the external right jugular vein for the purpose of drug administration. Apnea was induced by the administration of 1 mg of rocuronium bromide to inhibit spontaneous breathing followed by extubation. The apnea times for the pulse pressures of 5, 10, 15, 20 and 25 mmHg were recorded. The mean arterial and systolic blood pressures were also recorded for a comparison with the pulse pressure. Coefficients of variation (CV, standard deviation divided by the mean value) were calculated. The pulse pressure with the lowest CV was defined as a circulatory collapse.

### Preparation of HbV

HbV was prepared under sterile conditions, according to previously reported methods with only slight modifications [12]. Hemogloin (Hb) was purified from outdated donated human blood provided by the Japanese Red Cross Society (Tokyo, Japan). First, Hb was stabilised using carbonylation (HbCO) and pasteurized (60 °C for 12 h) to inactivate any viruses. All unstable enzymes are also eliminated by this procedure. The obtained Hb solution was concentrated by ultrafiltration to 42 g/dL. Subsequently, pyridoxal 5’-phosphate (PLP; Sigma Chemical Co., St. Louis, MO) was added to the HbCO solution as an allosteric effector at a molar ratio of PLP/Hb tetramer = 1. The Hb solution with PLP was then mixed with lipids and encapsulated in vesicles. The lipid bilayer comprised 1,2-dipalmitoyl-*sn*-glycero-3-phosphatidylcholine, cholesterol, 1,5-*O*-dihexadecyl-*N*-succinyl-L-glutamate (Nippon Fine Chemical Co. Ltd., Osaka, Japan) and 1,2-distearoyl-*sn*-glycerol-3-phosphatidylethanolamine-*N*-PEG5000 (NOF Corp., Tokyo, Japan) at a molar ratio of 5:4:0.9:0.03, respectively. The encapsulated HbCO was converted to HbO_2_ by exposing the liquid membranes of HbV to visible light under an O_2_ atmosphere. HbV was dialysed in normal saline to adjust the Hb concentration to 10 g/dL. Finally, the preparation was completely deoxygenated for long-term storage. The physiochemical parameters of HbV are as follows; P_50_, 17 - 23 mmHg; 251 ± 81 nm particle diameter; and less than 10% metHb content. Immediately before administration, deoxygenated HbV was drawn into a 10-mL syringe (Terumo Co., Tokyo, Japan) and rapidly oxygenated by mixing with air.

### Main experimental design

**Fig. 1:**
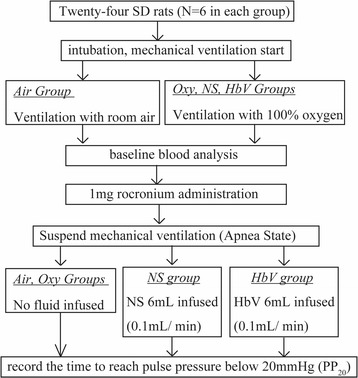
Outline of the experiment protocol. After the administration of rocuronium bromide, the mechanical ventilation was withdrawn to establish apnea. The apnea time to a pulse pressure less than 20 mmHg was compared between the groups

A schematic of the experimental design is shown in Fig. 1. Before the experiment, the rats were randomised into four groups (Air, Oxy, NS and HbV groups) and anaesthetised via the inhalation of 5% isoflurane (Mylan Inc. PA, USA) in 100% oxygen inside a plastic chamber. Once unconscious, tracheal intubation was performed using a 14-gauge intravenous catheter via direct laryngoscopy. The rats in the Air group were mechanically ventilated with 1.5% isoflurane in room air, while the rats in the other groups were ventilated with 1.5% isoflurane in 100% oxygen. All mechanical ventilation was performed using a small animal ventilator (Harvard Model 683, HARVARD APPARATUS, MA, USA). The respiratory rate was set to maintain pCO_2_ at 35–45 mmHg. The typical ventilator rates ranged between 55 and 65 breaths/min with a tidal volume of 8 mL/kg. The adequate anaesthesia depth was confirmed as described in the pilot study. Polyethylene catheters (SP45) filled with heparinised normal saline were inserted into the tail arteries to measure the arterial pressure. Polyethylene catheters filled with heparinised normal saline (SP55) were introduced via the right jugular vein into the right atrium for the purpose of drug infusion. Both types of polyethylene catheters were purchased from Natsume Seisakusho Co. Ltd. Tokyo, Japan. The blood pressure and pulse rate were monitored using a continuous monitoring system (PROPAQ 204 EL; Welch Allyn. NY, USA). The rectal temperature was maintained between 36.0 °C and 37.5 °C with the aid of thermal lighting during experiments. The baseline (3 min before insult) arterial blood analysis was performed (GEM Premiere 3000; Instrumental Laboratory, MA, USA) following the administration of 300 units of heparin to ensure that all values were within the normal range.

### Induction of apnea

A total dose of 1 mg rocuronium bromide (MSD. Inc., Tokyo, Japan) was administered while the animals were still under anaesthesia. The tracheal tubes were then removed and mechanical ventilation was withdrawn to establish apnea. The animals were visually observed to ensure that spontaneous breathing did not recover during the experiments. In addition, the rats in the Air and Oxy groups did not receive a fluid infusion during apnea. After a 30-s interval following ventilation withdrawal, the rats in NS and HbV groups received infusions of 6 mL normal saline or HbV, respectively, at a rate of 0.1 mL/s. The blood pressure and pulse rate were continuously recorded following the withdrawal of mechanical ventilation using a monitor display and video camera (iPad mini; Apple Inc., CA, USA). We defined a circulatory collapse as a pulse pressure less than 20 mmHg according to our preliminary study. The time to a pulse pressure less than 20 mmHg (PP_20_) was compared between the groups as the primary study outcome. Video recording was continued until the pulse pressure fell below 10 mmHg or until 180 s had expired. After the experiment, all rats were euthanised by an intravenous administration of 4 mEq potassium chloride.

### Randomisation and blinding of the experiment

The animals were randomly assigned to four groups before the induction of apnea. Randomisation was performed using a computer-generated randomisation table. The investigators who performed the experiments were not blinded since it was easy to distinguish which fluid was administered. The video recordings were investigated by a researcher who was blinded to the animal groups.

### Statistical analyses

Fully saturated Hemoglobin vesicles contain approximately 0.8 mL oxygen in a 6-mL solution according to the formula: Oxygen Content = 1.34 * [Hb] * [SaO_2_] = 1.34 * 10* 1.00. The oxygen consumption of adult rats is reported to be 0.68 mL/g/h (0.056 mL/s) [13]. We considered half of the time derived from the calculation (8 s) to be clinically significant (between group variance = 64). The within group variance calculated from the pilot study was 55.

A power analysis demonstrated that a group size of *n* = 5 was necessary to detect a difference in the mean PP_20_ values between the groups assuming a statistical power of 0.8 at a significance level of 0.05. We included six rats in each group to mitigate any loss due to accidental death or circulatory collapse during the study.

Means ± standard deviation are reported for all measurements unless otherwise specified. All of the data sets were analysed using Statflex software (Artech Inc., Tokyo, Japan). To reduce multiple comparisons, differences in the haemodynamic changes during apnea were compared at three time points (30, 90 and 150 s) by a repeated measure ANOVA followed by the Holm–Sidak test. Thirty seconds following the establishment of apnea was selected as the first time point as the fluid infusions were initiated at this time. As the fluids were infused for a duration of 60 s, the next time point used was 90 s, and a further time point at 150 s was chosen to maintain consistent intervals. Differences in the PP_20_ between each of the groups were analysed by a one-way ANOVA followed by the Holm–Sidak test. Since PP_20_ is a time-to-event variable, we also plotted it as a Kaplan-Meier curve with a log-rank analysis. The *P* values correspond to two-tailed tests with statistical significance set at *P* < 0.05.

## Results

### Pilot study to define circulatory collapse

The results of the ‘*Pilot study to define circulatory collapse*’ are presented in Table 1. We found that the apnoea time to a pulse pressure lower than 20 mmHg had the lowest CV value of 0.18. Therefore, we defined a pulse pressure lower than 20 mmHg as a surrogate outcome for the circulatory collapse in our main study.

**Table 1:** Study results of the apnea times to haemodynamic values

Blood pressure (mmHg)		Apnea time (s)		
		Mean	S.D.	CV
SBP	30	100.6	40.1	0.39
	25	113.8	42.1	0.36
	20	123.8	38.2	0.30
MAP	20	122.6	39.2	0.31
	15	143.8	33.9	0.23
	10	192.3	68.6	0.35
PP	25	26.6	7.9	0.29
	20	39.4	7.2	0.18
	15	46	9.4	0.20
	10	68.8	37.9	0.55
	5	78.8	41.2	0.52

Data are presented in seconds. A pulse pressure below 20 mmHg had the lowest CV (Coefficients of variation) and was used to define the circulatory collapse experimentally. All rats (*n* = 5) were mechanically ventilated with room air at 1 L/min and oxygen at 1 L/min to maintain FIO_2_ = 0.6

*SBP* systolic blood pressure; *MAP* mean arterial pressure; *PP* pulse pressure

### Baseline measurements

The initial body weight, preparation time and blood loss until apnea demonstrated small standard deviations within each group and did not differ significantly between groups. One rat in the Air group was excluded from the study due to a technical error. Therefore, all the results shown below are derived from 23 rats (*n* = 5 for the Air group; *n* = 6 for all the other groups).

The systolic blood pressure, mean arterial pressure, pulse pressure and pulse rate measurements at baseline for each group are shown in Table 2. The rats in the Air group exhibited a lower systolic blood pressure, mean arterial pressure and pulse pressure at baseline compared to the other groups. No significant difference in the systolic blood pressure, mean arterial pressure, or pulse pressure was observed between the NS, Oxy and HbV groups. The pulse rate at baseline was similar between all groups.

**Table 2:** Haemodynamic variables at baseline

	Air	Oxy	NS	HbV
SBP (mmHg)	67.4 (8.1)	99.8 (20.5)^a^	103.1 (13.7)^a^	113.1 (13.1)^a^
MAP (mmHg)	54.8 (5.8)	79.3 (15.9)^a^	84.5 (11.3)^a^	92.3 (11.7)^a^
PP (mmHg)	21.8 (5.1)	28.7 (4.5)^a^	28.1 (5.7)^a^	31.8 (4.1)^a^
PR (beats min^−1^)	421.6 (14.2)	450.5 (41.3)	410.8 (42.2)	425.0 (58.9)

There was no difference in haemodynamic variables between Oxy, NS and HbV groups at baseline. ^a^statistically significant difference from the Air group analysed by one-way ANOVA followed by a Holm-Sidak test (*P* < 0.01)

*SBP* systolic blood pressure; *MAP* mean arterial pressure; *PP* pulse pressure; *PR* pulse rate

The baseline blood analysis was performed prior to the induction of apnea. Table 3 summarises the baseline blood analyses. Rats in the Air group had significantly lower pO_2_ as they were ventilated with room air while the other groups were ventilated with 100% oxygen. All of the other variables were within the normal range with no differences were observed between groups.

**Table 3:** Baseline arterial blood analysis

	Air	Oxy	NS	HbV
pH	7.44 (0.04)	7.46 (0.03)	7.40 (0.09)	7.42 (0.08)
pO_2_ (mmHg)	73.4 (18.0)	399.1 (37.5)^a^	466.3 (53.9)^a^	433 (86.8)^a^
pCO_2_ (mmHg)	37.4 (2.2)	33.3 (3.4)	42.7 (17.1)	40.5 (7.3)
Hct (%)	40 (2.9)	39.5 (3.7)	38 (3.7)	37.8 (1.5)
Hb (g/dL)	12.4 (0.9)	12.5 (1.3)	11.8 (1.2)	11.8 (0.4)
Na (mmol/L)	135.4 (3.4)	134.0 (4.0)	134.3 (4.3)	133.2 (7.0)
K (mmol/L)	4.8 (0.5)	4.2 (0.5)	4.3 (0.8)	4.2 (0.5)
Ca (mmol/L)	1.2 (0.06)	1.2 (0.10)	1.2 (0.16)	1.2 (0.07)
BE (mmol/L)	1.62 (2.1)	−0.18 (1.7)	0.25 (2.9)	1.25 (2.4)

*Hct* haematocrit; *Hb* hemoglobin concentration (g/dL); *BE* base excess (mmol/L)

^a^Statistically significant difference between the Air group with a one-way ANOVA followed by a Holm-Sidak test (*P* < 0.01). Baseline arterial blood analysis was performed before the withdrawal of mechanical ventilation, and 0.3-mL arterial blood was collected from the tail artery. All variables except pO_2_ were within the normal range

### Haemodynamic changes during apnea

**Fig. 2:**
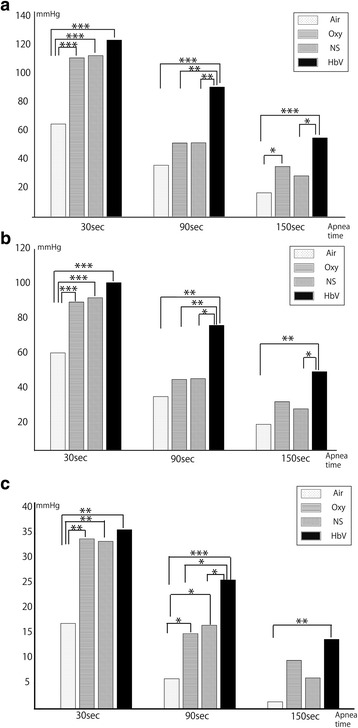
Haemodynamic changes after the withdrawal of mechanical ventilation. Systolic blood pressure (**a**), mean arterial pressure (**b**) and pulse pressure (**c**) for each group are shown. The rats in the Air group were ventilated with room air while the other groups were ventilated with 100% oxygen. The NS group were infused with 6 mL normal saline and the HbV group was infused with 6 mL HbV. The *arrows* indicate periods of fluid infusion. The differences between the groups were analysed by a one-way repeated measure ANOVA followed by a Holm-Sidak test. SBP, systolic blood pressure; MAP, mean arterial pressure; PP, pulse pressure. Symbols represent significant differences. *: *P* < 0.05; ** *P* < 0.01; *** *P* < 0.001

The systolic blood pressure, mean arterial pressure and pulse pressure measurements at 60 s intervals for each group are shown in Fig. 2. The systolic blood pressure, mean arterial pressure and pulse pressure was significantly lower in the Air group at 30 s compared to the other groups, while no differences were observed between the Oxy, NS and HbV groups. At 90 s, the systolic blood pressure, mean arterial pressure and pulse pressure were significantly higher in the HbV group compared to all other groups. At 150 s, the pulse pressure in the HbV group was higher compared to the Air group, while there were no significant differences between the Oxy and NS groups. However, the systolic blood pressure and mean arterial pressure in the HbV group were consistently significantly higher than the Oxy and NS groups.

### Apnea time to a pulse pressure less than 20 mmHg (PP_20_)

**Fig. 3:**
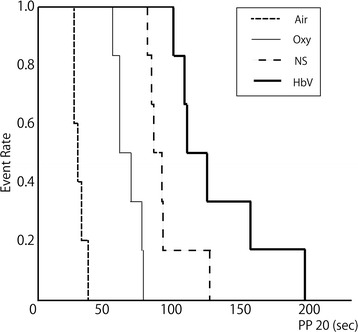
A Kaplan-Meier curve for the apnea time to a pulse pressure less than 20 mmHg for each group. There was a statistically significant difference between the groups assessed by a log-rank test (Air vs. HbV *P* = 0.004; Oxy vs. HbV, *P* = 0.003; NS vs. HbV, *P* = 0.045)

The apnea time to a pulse pressure less than 20 mmHg (PP_20_) was compared between the four groups as the primary outcome of this study. The mean PP_20_ values ± standard deviation were 30.4 ± 4.2 s, 67.5 ± 9.7 s, 95 ± 17.3 s and 135 ± 38.2 s for the Air, Oxy, NS and HbV groups, respectively. The mean PP_20_ values for all groups significantly differed from every other group by a one-way ANOVA followed by Holm-Sidak test (Air vs. Oxy *P* = 0.0001; Air vs. NS *P* = 0.0001, Air vs. HbV *P* = 0.002, Oxy vs. NS *P* = 0.002, Oxy vs. HbV *P* = 0.007 and NS vs. HbV *P* = 0.04). The Kaplan-Meier curves for the apnea time to a pulse pressure less than 20 mmHg are plotted in Fig. 3. A long rank analysis revealed statistical differences between the four groups. (Air vs. HbV *P* = 0.004; Oxy vs. HbV, *P* = 0.003; NS vs. HbV, *P* = 0.045). There were also similar trends regarding the time to reach pulse pressures below 15 [see Additional file 1] and 10 mmHg [see Additional file 2] between the groups.

## Discussion

To the best of our knowledge, this is the first report to demonstrate the effect of hemoglobin based oxygen carrier (HBOC) therapy during apnea. We found that the administration of oxygenated HbV more than doubled the apnea time to circulatory collapse when compared to the Oxy group.

In 1949, Amberson reported the first case series of clinical experiences using an Hb-saline solution [14]. Although it was effective for the delivery of oxygen, the Hb-saline solution was found to be associated with high renal toxicity and vasopressive reactions; therefore, its use was discontinued. Subsequently, a large variety of modified HBOCs have been developed in an attempt to overcome these complications. These include cross-linked polyHb, conjugated Hb, cross-linked tetrameric Hb, recombinant human Hb, and HbV [10, 11, 15–17]. A number of these modified HBOCs have also been used to treat anaemic patients for whom a blood transfusion is not readily available [18]. However, other reports have indicated that these products are still associated with significant toxic properties that have been attributed to myocardial infarction, acute renal failure, a deleterious increase in arterial blood pressure, and death [19, 20]. Such complications resulting from the use of HBOCs are thought to be mediated by the direct interaction between molecular Hb and the blood or endothelial cells; thus, preventing this direct contact via the encapsulation of Hb may lessen these complications. Previous studies have demonstrated that HbV does not exhibit renal toxicity or cause vasoconstriction [21]. Therefore, we can safely affirm that the haemodynamic difference observed in our study were not attributed to vasoconstriction.

Theoretically, an infusion of 6 mL HbV will prolong the apnea time to circulatory collapse by 15 s. To our surprise, the mean difference between the Oxy and the HbV groups was found to be 67.5 s. This discrepancy may be explained by the results from the NS group. The NS group was included to assess the effect of the infusion volume since HbV does not have collioid osmotic pressure. We predicted that an infusion greater than 30% of the total circulating volume would lead to acute heart failure, shortening the PP_20_ in the NS group. However, the PP_20_ in the NS group was higher than that of the Oxy group. One reason for this might be the blood viscosity. Infusing normal saline causes haemodilution, resulting in a better cardiac contraction in the NS and HbV groups. Recently, it has been demonstrated that a saline infusion, while increasing the left ventricular end diastolic volume, also decreases the left ventricular end systolic volume resulting in increased cardiac contraction [22]. Although the administration of normal saline may have an impact on the time to circulatory collapse, any negative effects (e.g. high chloride acidosis or hypernatremia) should be noted. Another possible explanation involves a higher level of effective oxygen transport by HbV than RBCs to the myocardium, as it has a high oxygen consumption and steep oxygen tension gradient. Since HbV is smaller than RBCs, it is distributed closer to the endothelial cell layer during arteriolar blood flow, whereas RBCs flow closer to the axial line [23]. Biochemical data (e.g. base excess or lactate levels) may have been informative and supported our analyses; however, blood samples from the tail artery during circulatory collapse were not obtained.

We used the pulse pressure to define circulatory collapse in our study. The apnea time to reach a pulse pressure less than 20 mmHg had the lowest coefficient of variation (CV); a low CV value indicates high reproducibility. Although the pulse pressure at baseline in our results was close to 20 mmHg, all of the animals exhibited an increased pulse pressure and systolic blood pressure, reflecting a hyper haemodyanamic state following apnoea. Moreover, this phenomenon is consistantly observed in this model [9], indicating that the actual pulse pressure decreased from a higher value (i.e. 40 mmHg). Finally, we chose a pulse pressure less than 20 mmHg as an outcome of this study based on our predefined protocol; however, there were also similar trends regarding the time to reach pulse pressures below 15 and 10 mmHg between the groups.

Finally, the infusion of oxygenated hemoglobin (artificial or not) will increase the total amount of oxygen in the body. Therefore, our experimental results are natural in most part. However, HbV is an artificial RBC, which can be stored at room temperature for more than 2 years. Moreover, the time required for the oxygenation of HbV in our experiment was within 5 s. During critical hypoxic situations (e.g. CVCI), the time required for the preparation of the drug would be essential.

### Limitations

Although all experiments were performed as thoroughly as possible, there are some limitations to this study. Firstly, we used the tail arteries to monitor any haemodynamic changes. As the tail artery is a small peripheral artery, the pulse pressure may be affected more by factors, such as blood pressure, blood volume and cardiac contractility. Thus, it may not be entirely reflective of the central haemodynamics, especially when the rats were in a state of near circulatory collapse. We have added one additional experiment to overcome this limitation, in which we monitored the pulse pressure in the tail artery and left common carotid artery consecutively. We found a strong relationship between the pulse pressures; however, they deviated greatly after the pulse pressure in the tail artery dropped lower than 25 mmHg. This may account for the finding that the CV was the lowest for 20 mmHg but increased for the lower pulse pressures. In addition, the femoral artery or descending aorta may have more accurately assessed the central circulation in this study.

Secondly, our results may have been confounded by the significantly lower haemodynamic variables in the Air group compared to other groups at baseline. However, we do not believe this to be a serious limitation of our study as we successfully achieved the main endpoint by demonstrating that the HbV group exhibited a prolonged time to collapse compared to the Oxy and NS groups.

## Conclusion

The infusion of HbV during apnoea in rats prolonged the time to circulatory collapse. The demonstration of HbV utility in a state of apnoea suggests that HbV may be efficacious as a bridging therapy in CVCI situations encountered clinically.

## Additional files

Additional file 1: Figure S1. A Kaplan-Meier curve for the apena time to a pulse pressure less than 15 mmHg for each group is shown. The PP_15_ times were 31.2 ± 5.1 s, 78.8 ± 21.0 s, 121 ± 53.8 s and 155 ± 44.4 s for the Air, Oxy, NS and HbV groups, respectively. Log-rank test revealed statistical difference between HbV and Air (*P* = 0.006), HbV and Oxy (*P* = 0.003) but did not differ between HbV and NS (*P* = 0.04). (PNG 133 kb)
Additional file 2: Figure S2. A Kaplan-Meier curve for the apena time to a pulse pressure less than 10 mmHg for each group is shown. The PP_10_ times were 58.8 ± 35.8 s, 136 ± 52.6 s, 155 ± 68.8 s and 190 ± 32.9 s for the Air, Oxy, NS and HbV groups, respectively. Log-rank test revealed statistical difference between HbV and Air (*P* = 0.006), HbV and Oxy (*P* = 0.04) but did not differ between HbV and NS (*P* = 0.82). (PNG 116 kb)

## Abbreviations

ANOVA: Analysis of variance
CV: Coefficients of variation
CVCI: Cannot ventilate, cannot intubate
Hb: Hemogloin
HBOC: Hb-based oxygen carrier
HbV: Hemoglobin vesicles
PLP: Pyridoxal 5’-phosphate
RBC: Red blood cell
