# Supplementary figures and images for: Hemoglobin Vesicles prolong the time to circulatory collapse in rats during apnea
Source: BMC Anesthesiol. 2017 Mar 14;17:44. doi: 10.1186/s12871-017-0338-y (PMC5348749; doi:10.1186/s12871-017-0338-y)

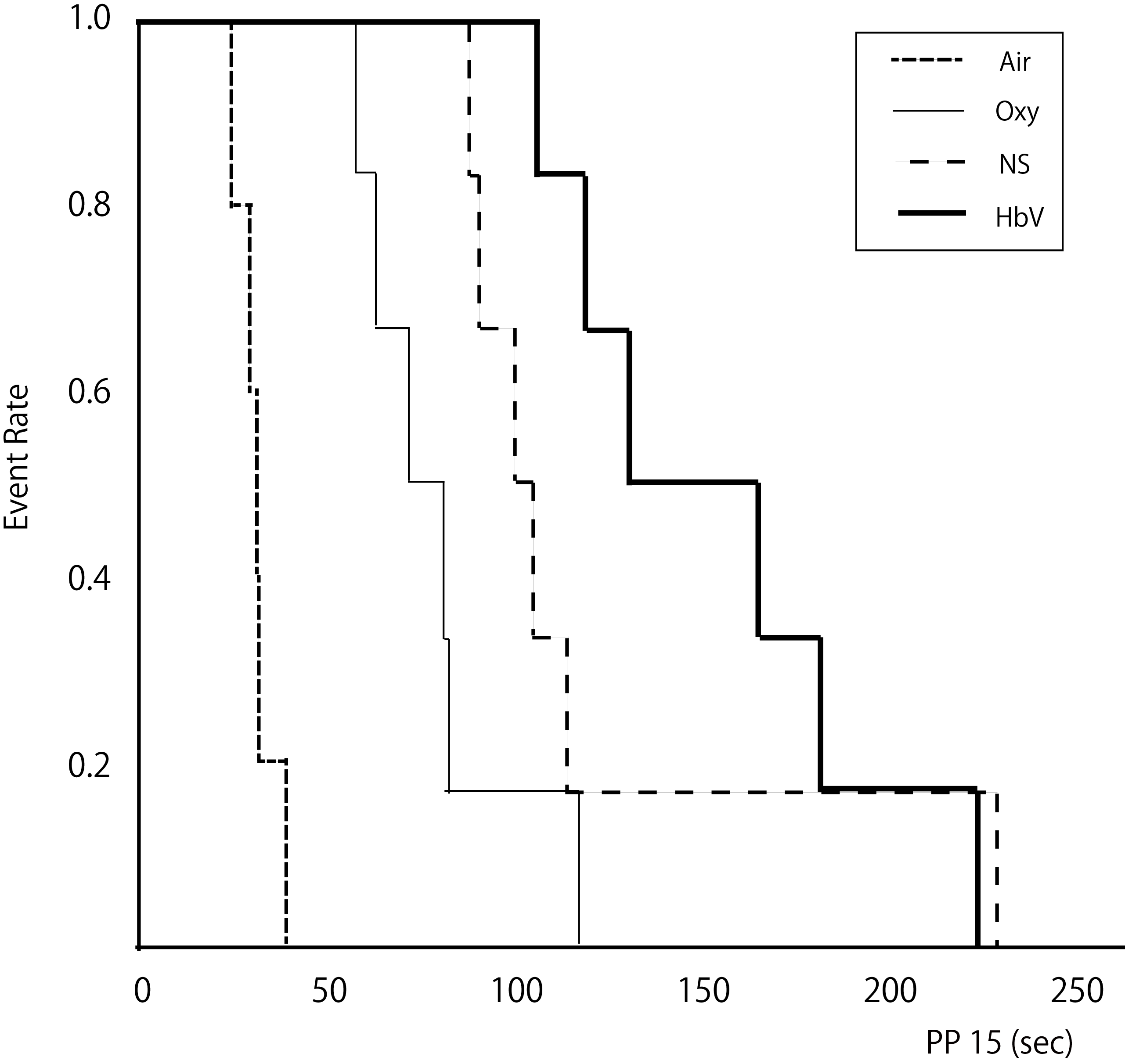

Supplement: Additional file 1: Figure S1. — A Kaplan-Meier curve for the apena time to a pulse pressure less than 15 mmHg for each group is shown. The PP15 times were 31.2 ± 5.1 s, 78.8 ± 21.0 s, 121 ± 53.8 s and 155 ± 44.4 s for the Air, Oxy, NS and HbV groups, respectively. Log-rank test revealed statistical difference between HbV and Air (P = 0.006), HbV and Oxy (P = 0.003) but did not differ between HbV and NS (P = 0.04). (PNG 133 kb) [file 12871_2017_338_MOESM1_ESM.png]

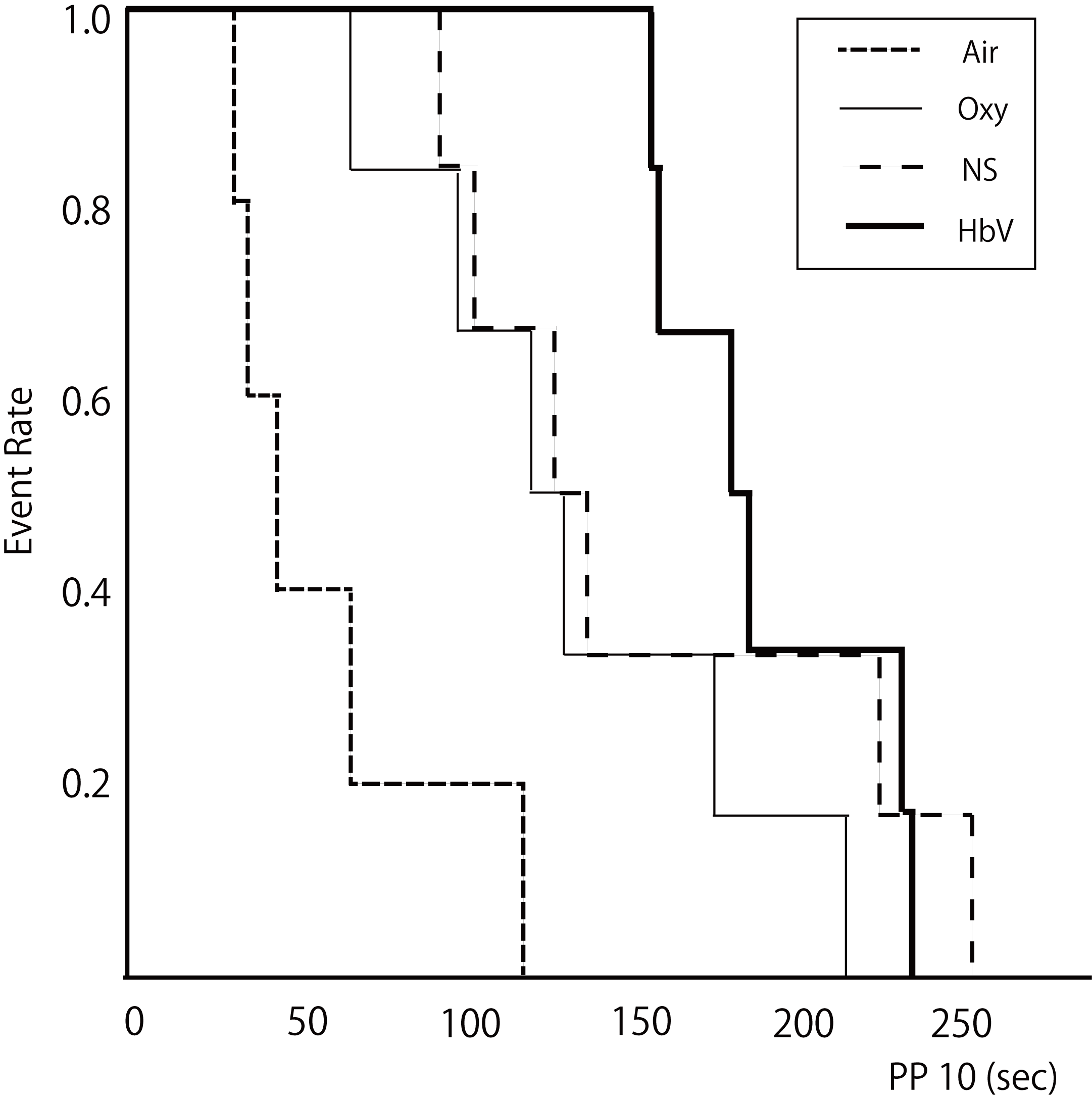

Supplement: Additional file 2: Figure S2. — A Kaplan-Meier curve for the apena time to a pulse pressure less than 10 mmHg for each group is shown. The PP10 times were 58.8 ± 35.8 s, 136 ± 52.6 s, 155 ± 68.8 s and 190 ± 32.9 s for the Air, Oxy, NS and HbV groups, respectively. Log-rank test revealed statistical difference between HbV and Air (P = 0.006), HbV and Oxy (P = 0.04) but did not differ between HbV and NS (P = 0.82). (PNG 116 kb) [file 12871_2017_338_MOESM2_ESM.png]
